# Distinguishing naive‐ from memory‐derived human B cells during acute responses

**DOI:** 10.1002/cti2.1090

**Published:** 2019-11-13

**Authors:** Maria Auladell, Thi Ho Nguyen, Beatriz Garcillán, Fabienne Mackay, Katherine Kedzierska, Annette Fox

**Affiliations:** ^1^ Department of Microbiology and Immunology University of Melbourne at the Peter Doherty Institute for Infection and Immunity Melbourne VIC Australia; ^2^ WHO Collaborating Centre for Reference and Research on Influenza VIDRL at the Peter Doherty Institute for Infection and Immunity Melbourne VIC Australia

**Keywords:** B cell, differentiation, memory, monocyte, naive, TLR

## Abstract

**Objectives:**

A fundamental question in influenza research is whether antibody titre decline upon successive exposure to variant strains is consequent to recall of cross‐reactive memory B cells that competitively inhibit naive B‐cell responses. In connection, it is not clear whether naive and memory B cells remain phenotypically distinct acutely after activation such that they may be distinguished *ex vivo*.

**Methods:**

Here, we first compared the capacity of anti‐Ig and Toll‐like‐receptor (TLR) 7/8 and TLR9 agonists (R848 and CpG) to augment human B‐cell differentiation induced by IL‐21 and sCD40L. The conditions that induced optimal differentiation were then used to compare the post‐activation phenotype of sort‐purified naive and memory B‐cell subsets by FACS and antibody‐secreting cell (ASC) ELISPOT.

**Results:**

Sort‐purified naive and memory B cells underwent robust plasmablast and ASC formation when stimulated with R848, but not CpG, and co‐cultured with monocytes. This coincided with increased IL‐1β and IL‐6 production when B cells were co‐cultured with monocytes and stimulated with R848, but not CpG. Naive B cells underwent equivalent ASC generation, but exhibited less class‐switch and modulation of CD27, CD38 and CD20 expression than memory B cells after stimulation with R848 and monocytes for 6 days.

**Conclusion:**

Stimulation with R848, IL‐21 and sCD40L in the presence of monocytes induces robust differentiation and ASC generation from both naive and memory B‐cells. However, naive and memory B cells retain key phenotypic differences after activation that may facilitate *ex vivo* discrimination and better characterisation of acute responses to variant antigens.

## Graphical abstract

This study contrasts the requirements and outcomes of human B‐cell subset differentiation *in vitro*. R848 induced more potent B‐cell differentiation than CpG when combined with sCD40L and IL‐21, an effect that was mediated via monocyte activation by R848 but not by CpG. Naive and memory B cells retained key phenotypic differences after activation that may facilitate *ex vivo* discrimination and better characterisation of acute responses to variant antigens.

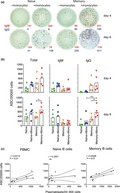

## Introduction

It is challenging to induce long‐term immunity against highly mutable viruses such as influenza viruses, not only due to immune escape, but also due to a propensity for antibody levels to decline with successive exposures to variant influenza virus strains. This phenomenon, first described in the 1950s, and referred to as original antigenic sin,^1^ may be due to memory B cells that cross‐react with shared epitopes in subsequent strains and outcompete naive B cells for the resources required for activation.^2^ There is great interest in understanding if, and when, memory B‐cell dominance occurs, and how it may influence antibody titre and breadth. However, there is a lack of simple methods to define whether activated human B cells detected *ex vivo* following antigen exposure were originally naive or memory B‐cells. Although resting memory and naive human B cells can be distinguished via phenotypic markers such as CD27 and CD21, it is unclear how rapidly markers change upon activation, and whether they can be distinguished phenotypically once activated. Therefore, this study examined how expression of key phenotypic markers changes after *in vitro* activation, and with division, of human peripheral blood naive and memory B‐cells.

We set out to use a stimulation protocol that maximises B‐cell differentiation into antibody‐secreting cells (ASCs), otherwise called plasmablasts, in order to mimic a robust *in vivo* response. It is increasingly apparent that robust B‐cell differentiation requires innate Toll‐like‐receptor (TLR) signals, adaptive BCR signals and T cell helper signals such as IL‐21 and CD40L.^3^, ^4^, ^5^, ^6^, ^7^, ^8^, ^9^ Similarly, it has been established that B‐cell subsets will not differentiate in the absence of non‐B cells.^9^, ^10^ Agonists of TLR7/8 (R848) and TLR9 (CpG) induce similar gene expression in human B‐cells.^11^ R848 and, to a lesser extent, CpG are also sufficient to induce differentiation of memory B‐cells, but not of naive B‐cells.^12^, ^13^ Studies comparing the ability of R848 and CpG to augment B‐cell stimulation via BCR and T‐cell signals are lacking, as are protocols to induce robust naive B‐cell differentiation. Therefore, we compared B‐cell and B‐cell subset differentiation following *in vitro* stimulation with R848 versus CpG, both combined with IL‐21 and sCD40L, and tested with and without anti‐Ig, which targets BCR signalling pathways. These stimuli, in particular R848, induced robust B‐cell differentiation when using PBMCs but not when using purified B‐cell subsets cultured with non‐B lymphocytes. We therefore stimulated purified B‐cell subsets in cultures containing monocytes as well as non‐B lymphocytes and observed robust differentiation using a combination of R848, IL‐21 and sCD40L without anti‐Ig. Having established a protocol for robust *in vitro* B‐cell differentiation, we compared the phenotype of naive and memory B cells after activation. We detected key differences in surface marker expression at early time points after activation that may facilitate discrimination of naive‐ from memory‐derived B cells in human samples collected early after antigen exposure.

## Results

### Human B‐cell stimulation via TLR7/8 induces greater differentiation than stimulation via TLR9

**Figure 1:**
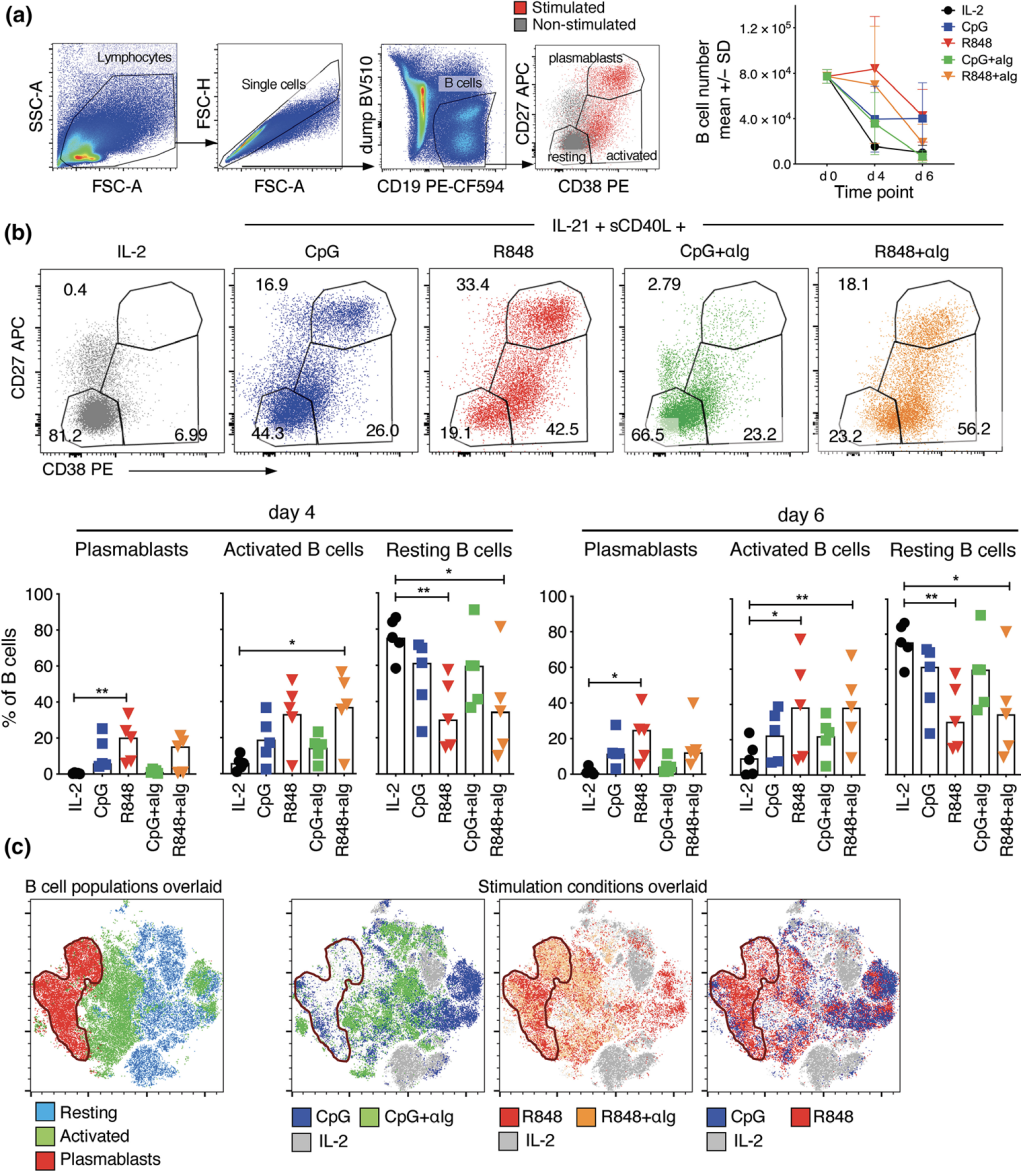
Comparison of stimuli for human B‐cell activation. PBMCs from five donors were cultured with IL‐21 and sCD40L combined with either CpG or R848 alone, or also with anti‐human Ig for 4 and 6 days before quantifying the proportions that had undergone differentiation. Two PBMC sets were freshly isolated (and generally responded better across all the stimuli), while the remaining PBMCs were not processed immediately after collection. **(a)** B‐cell analysis gates and number of PBMCs that are B cells before and 4 and 6 days after culture. CD19^+^CD3^−^CD14^−^CD16^−^ B cells were classified as plasmablasts or resting or activated cells based on CD27 and CD38 expression by stimulated (coloured) versus unstimulated (grey) B cells. FACS plots show results for a representative donor on day 4. **(b)** Graphs show percentages of B cells in the three analysis gates for individual donors (symbols, *n* = 5) as well as medians (bars). Asterisks indicate stimuli that had a significant effect on percentages compared to the IL‐2 control using Friedman test, *P < 0.05, **P < 0.01. **(c)** tSNE plots showing clustering of cells within the B‐cell gate of a representative donor on day 6, concatenating data for all stimulation conditions, and overlaying either the pre‐defined B‐cell analysis gates based on CD27 and CD38 expression (left panel) or the stimulation conditions (right panels).

While both TLR7/8 and TLR9 agonists can augment B‐cell differentiation induced by CD40L and IL‐21, it is not clear which is superior, or whether they should be combined with each other or with anti‐Ig to co‐stimulate B cells via the BCR. To address these questions, we cultured total PBMCs from five healthy human donors with sCD40L and IL‐21 and either CpG or R848, both of which were tested with and without antigen‐binding fragments (F(ab’)2) of anti‐human Ig. All cultures contained IL‐21 and sCD40L, so hereafter stimuli are referred to as simply CpG, R848, CpG+anti‐Ig or R848+anti‐Ig. In preliminary studies, we also stimulated PBMCs with a combination of CpG and R848 and found no enhancement of B‐cell differentiation compared to R848 alone (Supplementary figure 1). Flow cytometry was performed on days 4 and 6 to classify CD19^+^ B cells as CD27^hi^CD38^hi^ plasmablasts, or CD27^+/−^CD38^+^ activated or CD27^−^CD38^−^ resting B cells in comparison with non‐stimulated (IL‐2 only) cultures (Figure 1a). Plasmablasts were substantially enriched at both time points in all stimulated cultures except CpG+anti‐Ig (Figure 1a and b). Similarly, activated B cells were enriched and resting B cells were depleted in all stimulated cultures except CpG+anti‐Ig. R848 was the most potent of the stimuli used in terms of the percentages of B cells with activated and plasmablast phenotypes (Figure 1b) as well as the absolute numbers of activated B cells and plasmablasts (Supplementary figure 2a). Plasmablast numbers declined from day 4 to day 6 (Supplementary figure 2a), consistent with a drop in total B‐cell number (Figure 1a, top right panel), which was probably due to B‐cell death. BCR stimulation with anti‐Ig did not augment differentiation induced by R848 or CpG.

Stimulation conditions were further compared using t‐distributed stochastic neighbour embedding (tSNE) to integrate data for all surface markers assessed (CD19, CD20, CD21, CD27, CD38, CD71, IgM, IgG, IgD) as well as forward and side scatter (Figure 1c). B cells clustered according to differentiation phenotype, validating the classification based on CD27 and CD38 expression (Figure 1c, left panel). More importantly, B cells clustered according to the presence and type of stimulation (Figure 1c, right panels), providing further evidence that R848 and CpG have different capacities to induce B‐cell differentiation and that anti‐Ig impacts both. R848‐stimulated cells largely clustered in the same region as B cells with activated and plasmablast phenotypes; CpG+anti‐Ig‐stimulated cells largely clustered in the same region as B cells with resting phenotypes, but in distinct clusters from non‐stimulated cells; and CpG‐stimulated B cells largely clustered in the same region as B cells with an activated phenotype. The greater capacity of R848 compared to CpG to stimulate B‐cell differentiation was further demonstrated by more frequent detection of ASCs by ELISPOT (Supplementary figure 2b).

Taken together, these results demonstrate clear superiority of R848 compared to CpG for inducing B‐cell differentiation *in vitro*, and that the addition of anti‐human Ig does not further enhance differentiation.^14^

### Robust differentiation of naive and memory B cells when stimulated in the presence of monocytes

**Figure 2:**
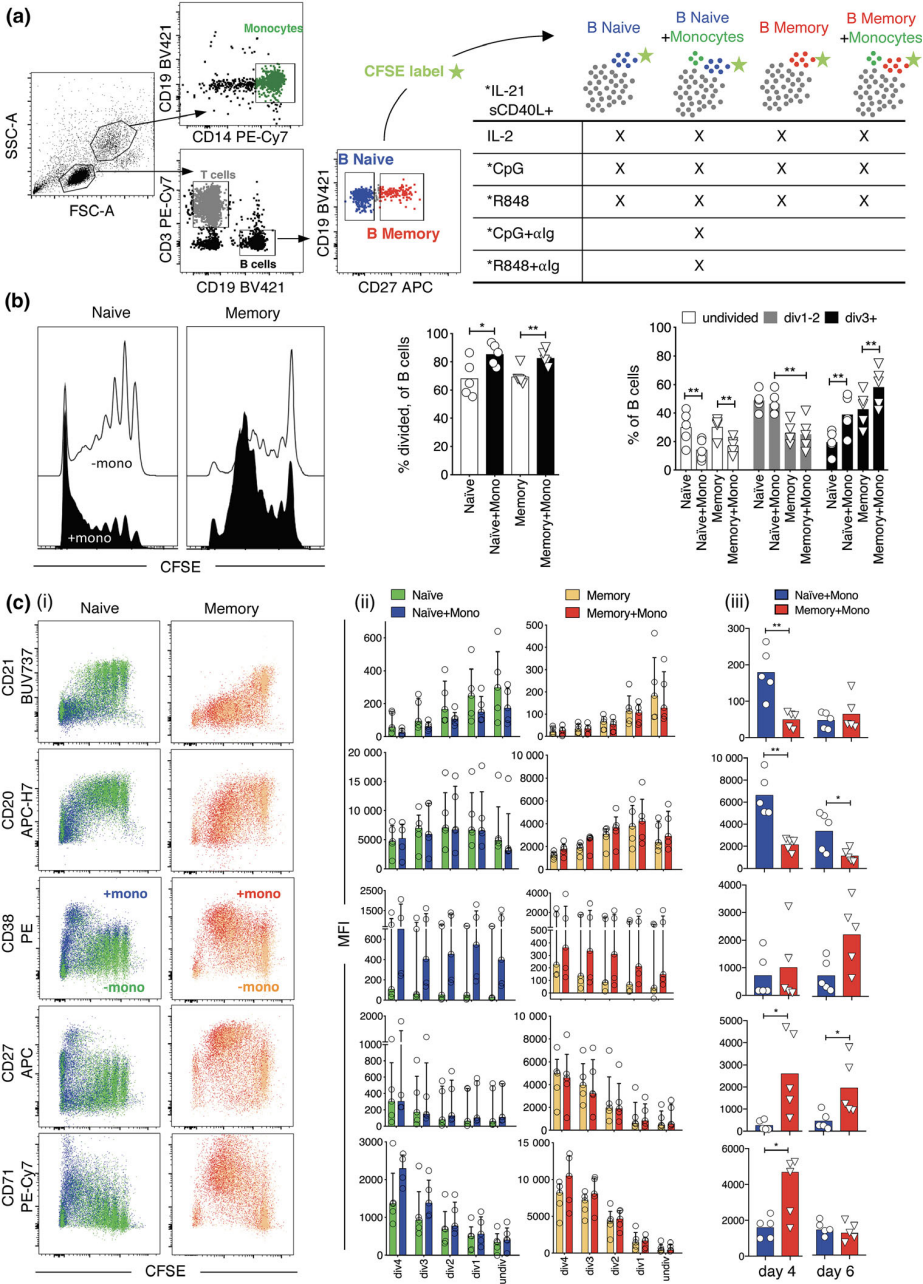
B‐cell subset division and differentiation are enhanced when stimulated in the presence of monocytes. (**a**) Experimental plan for cell sorting, mixing and stimulation. (**b**) B‐cell subset division after stimulation with R848 without and with monocytes. Results for day 6 (naive B‐cells) or day 4 (memory B‐cells) are shown for a representative donor and summarised for individual donors (*n* = 5) as percentage of B cells that have divided (middle panels), and percentages that reached division at least 1 or 3 divisions (right panel). Results are presented as symbols for each donor and as the mean of all donors (bars). Asterisks indicate significance by paired *t*‐test, *P < 0.05, **P < 0.01. (**c**) Surface marker expression change with each division after R848 stimulation without and with monocytes. (**ci**) Results for naive B cells on day 6 and memory B cells on day 4 are shown for a representative donor. (**cii**) Mean fluorescence intensities (MFIs) at each division are shown for each donor (symbols) and summarised as medians and interquartile ranges. (**ciii**) MFIs for each marker are compared for naive versus memory B‐cells irrespective of division on days 4 and 6 after stimulation with R848 in the presence of monocytes. Results are shown for individual donors (symbols) and as the median of all donors. Asterisks indicate significant difference between naive and memory B cells by Wilcoxon test. Results of five different donors from three independent experiments.

To compare the phenotypes of human CD27^−^ naive versus CD27^+^ memory B cells after activation, subsets were sorted based on CD27 expression, mixed with sorted non‐B lymphocytes, containing mainly T cells (Figure 2a, left panel), and then stimulated with R848, IL‐21 and CD40L. However, in our initial experiments, differentiation of naive and memory B cells was poor compared to total PBMCs from the same donor (data not shown). We hypothesised that monocytes may also be required for B‐cell differentiation since they were lacking from the B‐cell subset cultures. To examine this, monocytes, defined to be CD14^+^ cells with high forward and side scatter compared to lymphocytes, were sorted and added to half of the cultures containing sorted naive or memory B cells and non‐B lymphocytes (Figure 2a, right panel). In each culture, B cells represented 10% of the total cultured cells and, when present, monocytes represented 5% to mimic the proportions found in human blood. Significantly more naive and memory B cells entered division, observed via a reduction in CFSE staining, and progressed to division 3 when stimulated in the presence of monocytes (Figure 2b). In addition, phenotypic changes were more pronounced at earlier divisions when B cells were stimulated with monocytes (Figure 2ci and ii). In particular, CD38 was expressed by both naive and memory B‐cells prior to division when stimulated with monocytes present, and levels remained higher throughout divisions than in B cells stimulated without monocytes. Although CD27 expression increased with division, expression per division was not notably enhanced by adding monocytes. CD71 expression increased with division for both subsets and all conditions consistent with its use as a marker of dividing cells.

Having established that monocytes augment B‐cell division and differentiation, we then compared surface marker expression between naive and memory B cells after stimulation in the presence of monocytes (Figure 2ciii). On days 4 and 6, naive‐derived B cells expressed substantially lower levels of CD27 and, to a lesser extent CD38, significantly higher levels of CD20 than memory‐derived B cells. CD71 expression remained high on day 6 relative to the unstimulated non‐dividing cells, indicating that this may be a useful marker for *ex vivo* identification of B cells involved in acute responses (Supplementary figure 3). Therefore, based on our selective panel of differentiation markers, we could potentially distinguish recently activated naive B‐cells (i.e. CD27^low^, CD38^low^, CD20^high^) from activated memory‐derived B cells (i.e. CD27^high^, CD38^high^, CD20^low^) *ex vivo* when assessing acute responses to infection or vaccination.

### Monocytes enhance plasmablast generation from naive and memory B cells when using R848 but not CpG

**Figure 3:**
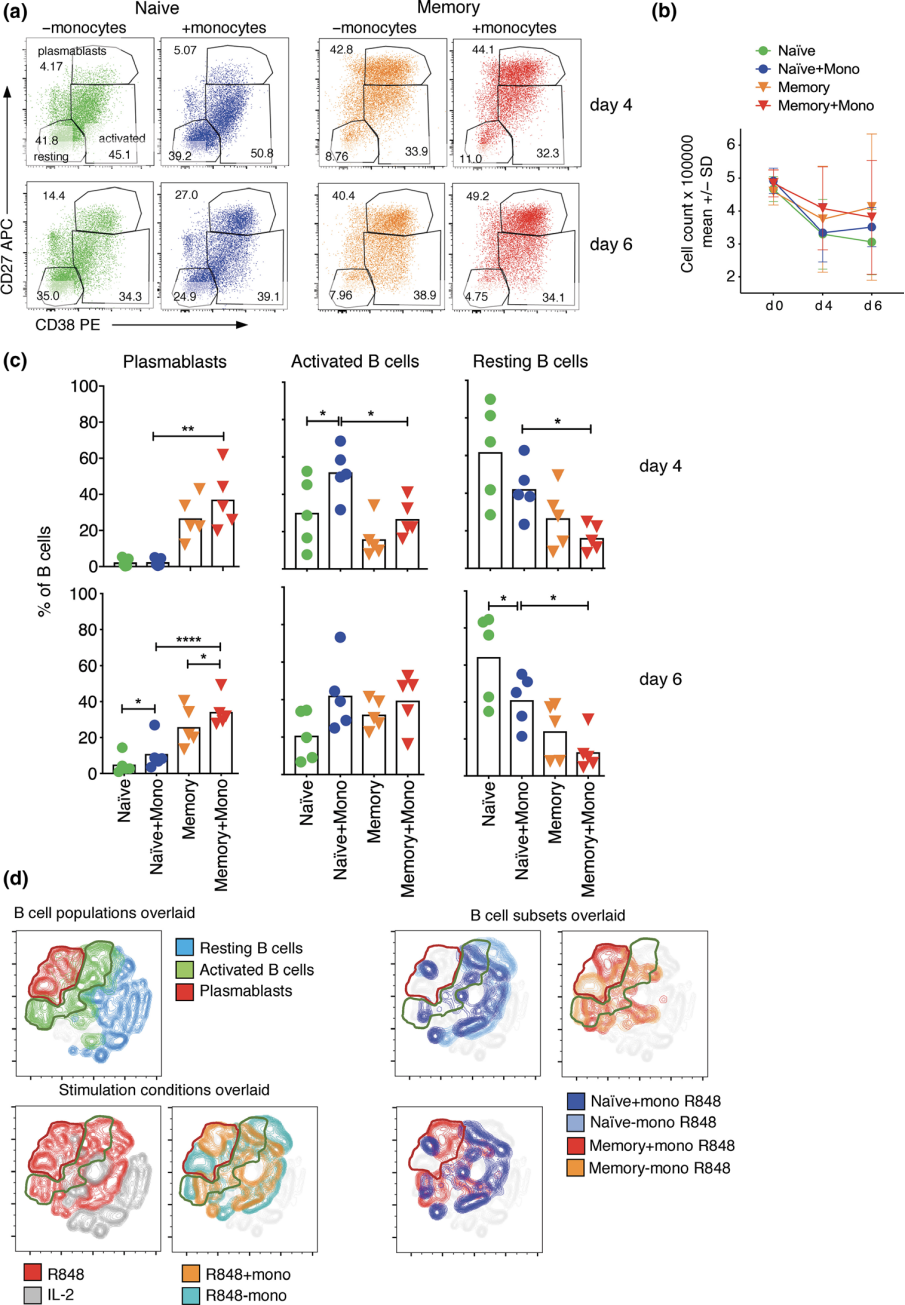
Plasmablast formation is enhanced when B‐cell subsets are stimulated with R848 and co‐cultured with monocytes. (**a**) Count of cells in the cultures before and after stimulation with and without monocytes. (**b**) FACS profiles of B cells from a representative donor that have been stimulated without and with monocytes for 6 days (naive B‐cells) or 4 days (memory B‐cells). (**c**) Percentages of naive and memory B‐cells in the three analysis gates after stimulation with R848 are shown for individual donors (symbols, *n* = 5), and as means for all donors (bars) with asterisks indicating a significant effect of monocytes using paired *t*‐test, *P < 0.05, **P < 0.01, ****P < 0.0001. (**d**) tSNE plots show clustering of cells within the B‐cell gate based on all markers assessed and light scatter. Results are shown for a representative donor on day 6, concatenating data for all stimulation conditions and both B‐cell subsets, then overlaying either the original analysis gates (left panel), the different stimulation conditions (middle panels) or the different subsets stimulated with and without monocytes (right panels). Results of five different donors from three independent experiments.

The capacity for monocytes to augment B‐cell differentiation stimulated by TLR agonists was further explored by measuring plasmablast generation via flow cytometry and ELISPOT. We also investigated whether monocytes augment B‐cell differentiation stimulated by CpG, and the effects of adding anti‐Ig to B cells stimulated in the presence of monocytes. As expected, monocyte addition resulted in increased plasmablast formation (Figure 3a and c). Monocyte addition had minimal effect on cell numbers recovered (Figure 3b), indicating that plasmablast accumulation is a result of increased B‐cell division and differentiation rather than to any potential change in starting number or proportion of cells that are B cells when monocytes are added. tSNE plots encapsulating all markers assessed showed distinct clustering of R848‐stimulated B cells with and without monocytes (Figure 3d, bottom left panels), and that B cells stimulated with monocytes predominate in regions where cells with activated and plasmablast phenotypes cluster (Figure 3d, top left panel). While plasmablasts were readily generated from R848‐stimulated memory B cells without monocytes (Figure 3c), it was interesting that they clustered differently from plasmablasts generated with monocytes (Figure 3d, top right panels). Of importance, naive and memory B cells clustered differently, even when stimulated in the same way, and within the region defined by cells with a plasmablast phenotype (Figure 3d, top right panels). Indeed, when analysis was limited to cells stimulated with R848 in the presence of monocytes, naive and memory B cells clustered completely separately on day 4 (Supplementary figure 4). While there was more overlap of naive and memory clusters on day 6, they remained largely distinct, even within the plasmablast region (Figure 3d, bottom right panel).

**Figure 4:**
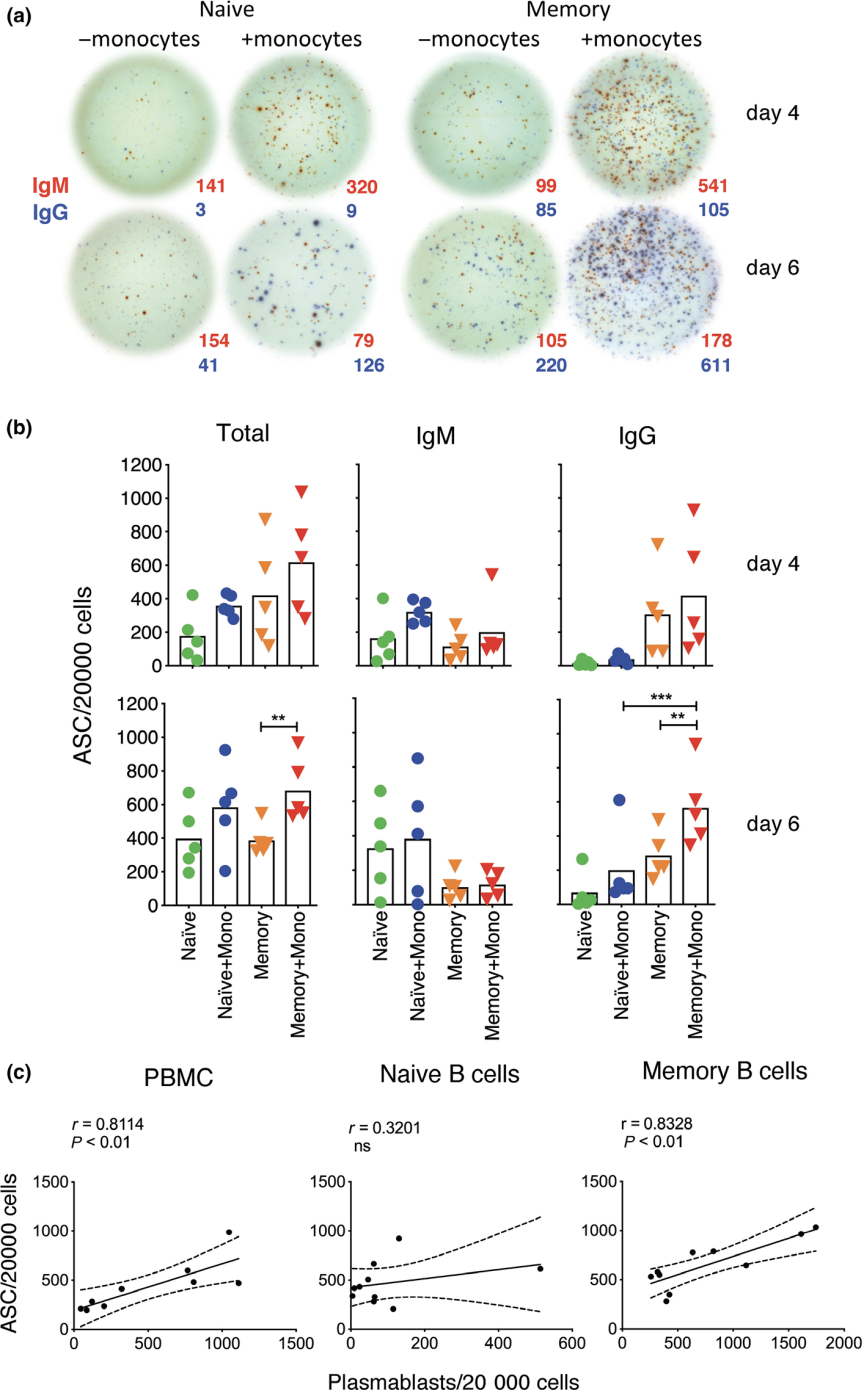
Increased detection of total and class‐switched ASCs when naive and memory B cells are stimulated in the presence of monocytes. (**a**) ASC ELISPOT images of naive and memory B cells from a representative donor that have been stimulated with sCD40L, IL‐21 and R848 for 4 and 6 days with and without monocytes and then incubated on ELISPOT plates for 5 h to detect cells secreting IgM (red spots) and IgG (blue spots). (**b**) Numbers of total, IgM and IgG ASCs are shown for individual donors (symbols, *n* = 5), and as means for all donors (bars). Asterisks indicate significant effects of monocytes assessed using paired *t*‐test, **P < 0.01, ***P < 0.001. Results of five different donors from three independent experiments.

The ability of monocytes to enhance B‐cell differentiation was further demonstrated by dual‐colour ELISPOT, which showed that numbers of total and class‐switched ASCs increased when monocytes were added (Figure 4). It was somewhat surprising that the overall frequency of ASCs was nearly as high in naive as in memory B‐cell cultures by day 6 (Figure 4b) because significantly fewer naive than memory B cells developed a plasmablast phenotype (Figure 3c). Accordingly, frequencies of phenotypically defined plasmablasts and ASCs correlated well for PBMCs and memory B‐cells, but not naive B‐cells (Figure 4c), indicating that at least some naive‐derived ASCs do not acquire a typical plasmablast phenotype.

**Figure 5:**
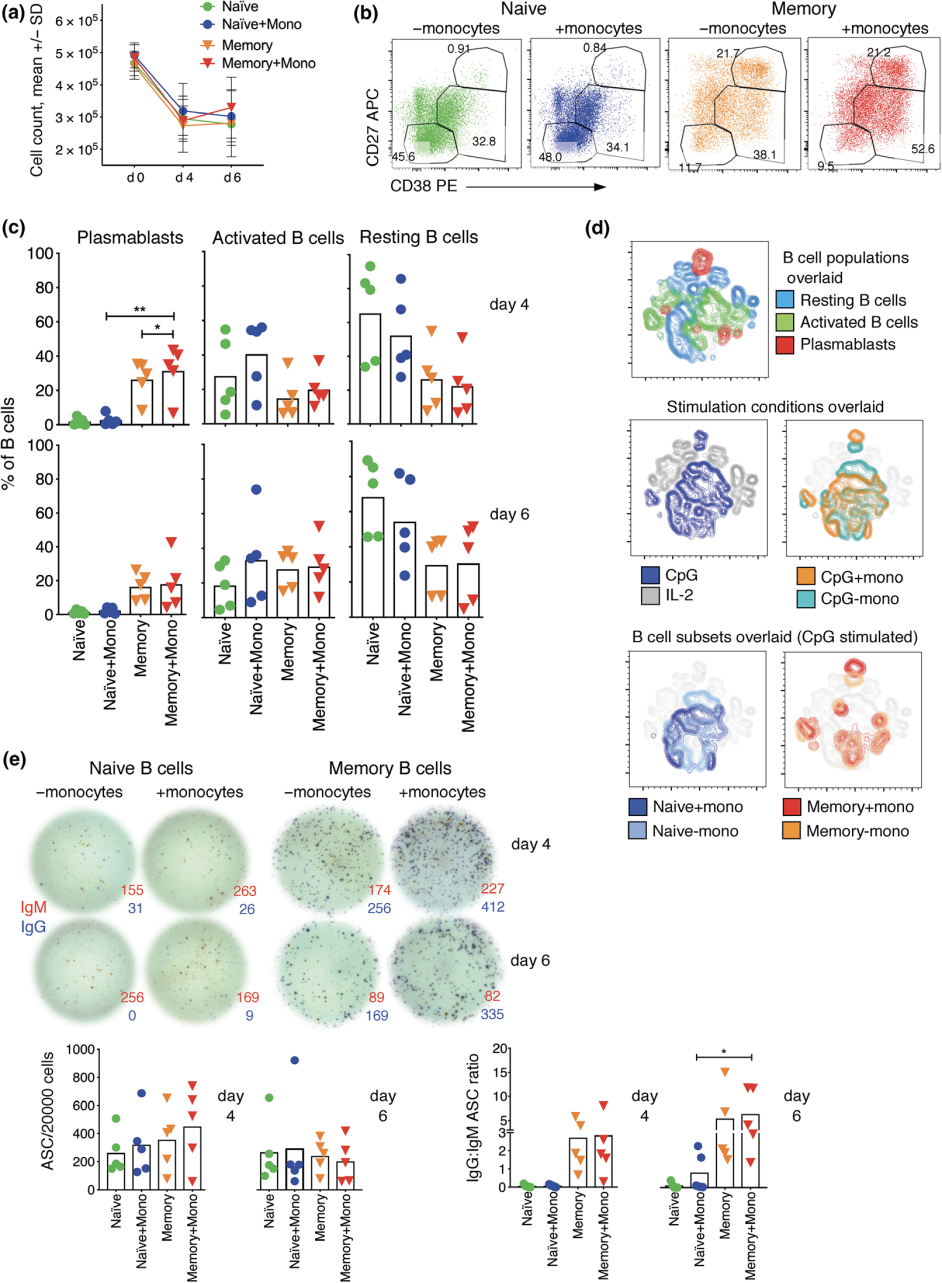
Monocytes do not enhance B‐cell subset differentiation when stimulated with CpG. (**a**) Count of cells in the cultures before and after stimulation with CpG. (**b**) FACS profiles of B cells from a representative donor that have been stimulated with sCD40L, IL‐21 and CpG, with and without monocytes for 4 days. (**c**) Percentages of B cells in the three analysis gates defined by CD27 and CD38 expression showing values for individual donors (symbols, *n* = 5) and means for all donors (bars). Asterisks indicate significance using paired *t*‐test, *P < 0.05, **P < 0.01. (**d**) tSNE plots indicate clustering of cells within the B‐cell gate of a representative donor assessed on day 6, concatenating data with and without CpG and/or monocytes, and overlaying B‐cell analysis gates based on CD27 and CD38 expression (top panels), stimulation conditions (middle panels) and the two subsets stimulated with and without monocytes (bottom panels). (**e**) Effect of monocytes on ASC detection after stimulation with CpG, as in Figure 3. ELISPOT images are shown for a representative donor, and ASC numbers, as well as ration of IgG to IgM ASCs, are shown for five donors. Asterisks indicate significance, as above. Results of five different donors from three independent experiments.

Interestingly, monocyte addition had little or no effect on naive or memory B‐cell differentiation after stimulation with CpG, based on both phenotypic analysis (Figure 5a–d) and ASC detection by ELISPOT (Figure 5e). Anti‐Ig prevented naive B cells from forming plasmablasts or ASCs after stimulation with R848 or CpG (Supplementary figure 5), consistent with effects of anti‐Ig on PBMCs. Notably, cell division was markedly enhanced by the addition of anti‐Ig (Supplementary figure 5), contrary to the tendency for differentiation to be linked to division.

Taken together, these results confirm that monocytes enhance *in vitro* B‐cell differentiation induced by R848, but not CpG, and that naive B cells remain phenotypically distinct from memory B cells for at least 6 days after stimulation, and even after differentiating into plasmablasts.

### Naive B cells undergo limited differentiation when co‐cultured with monocytes alone compared to monocytes and T cells, whereas memory B‐cell differentiation is enhanced when T cells are lacking

**Figure 6:**
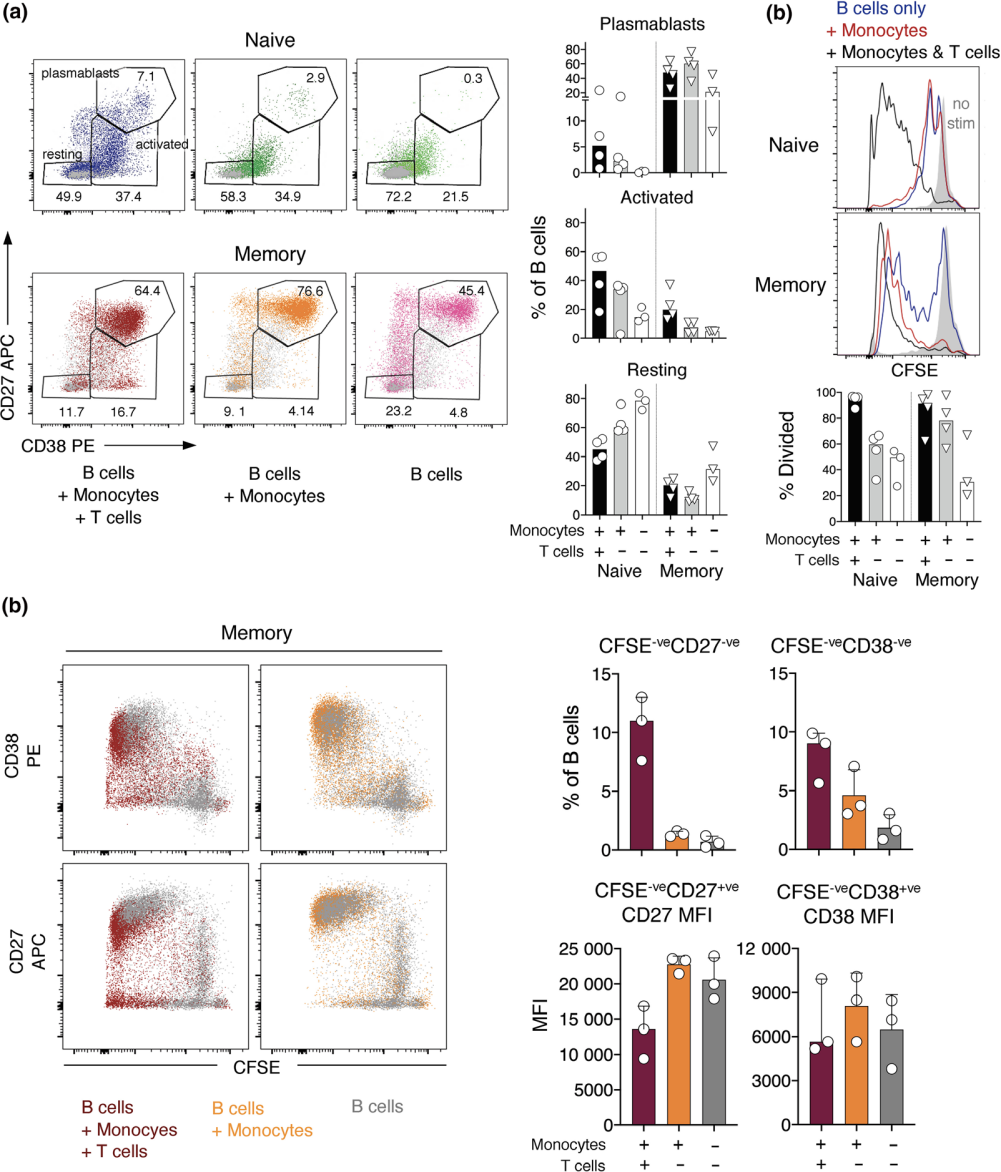
Monocytes augment B‐cell subset differentiation after stimulation with R848 in the absence of T cells, most notably for memory B‐cells. (**a**) Left panel: FACS profiles of B cells from a representative donor that have been stimulated with monocytes and T cells, with monocytes only, or on their own for 6 days. Grey dots indicate equivalent cell mixtures cultured without stimulation. Right panel: percentages of naive and memory B cells in the three analysis gates after stimulation with R848 are shown for individual donors (symbols, *n* = 4 when monocytes are present, and *n* = 3 when B cells are cultured alone), and as medians for all donors (bars). (**b**) B‐cell subset division after stimulation with R848 without and with monocytes and/or T cells. Results are presented as in **(a)**. (**c**) Left panel: surface marker expression change with each division is shown for memory B cells from a representative donor after 6 days of R848 stimulation without and with monocytes and/or T cells. Right panel: CD27 and CD38 expression is shown for memory B cells that have divided to become CFSE^−^ after 6 days of culture without and with monocytes and/or T cells. Results are shown for individual donors (symbols, *n* = 3), and as medians and interquartile ranges.

Given the ability of monocytes to increase B‐cell differentiation, we then assessed whether B‐cell subsets undergo differentiation or division when stimulated with sCD40L, IL‐21 and R848 in the presence of monocytes without non‐B lymphocytes. The proportion of naive B cells with activated and plasmablast phenotypes increased modestly when monocytes were added but did not reach the levels detected when both monocytes and T cells were added (Figure 6a). In contrast, a greater proportion of memory B cells differentiated into plasmablasts when monocytes were added without T cells than with T cells (Figure 6a). Similarly, addition of monocytes alone was associated with substantial division of memory B cells but only modest division of naive B‐cells (Figure 6b). It was striking that most memory B cells develop a plasmablast phenotype when activated in the presence of monocytes alone and very few remain only partially activated (Figure 6a). This was not due to attenuation of division when T cells were added (Figure 6b), but rather to attenuation of CD27 and CD38 expression at later divisions, giving rise to more CFSE^−^, CD27^−^ and CD38^−^ memory B‐cells (Figure 6c). This suggests that the fate of memory B cells may depend on the balance of stimulation via adaptive‐T cell and innate‐monocyte/TLR pathways.

Taken together, the results indicate that a small proportion of naive B cells are capable of fully differentiating into plasmablasts when only monocytes are present in the system and that when memory B cells receive predominantly innate signals derived from monocytes, they fully differentiate into plasmablasts and hardly any cells remain CD27 and CD38 low or negative. Importantly, for both subsets, monocytes seem to play a key role inducing cell division and differentiation.

### Cytokine production in naive and memory B‐cell stimulation cultures with and without monocytes

**Figure 7:**
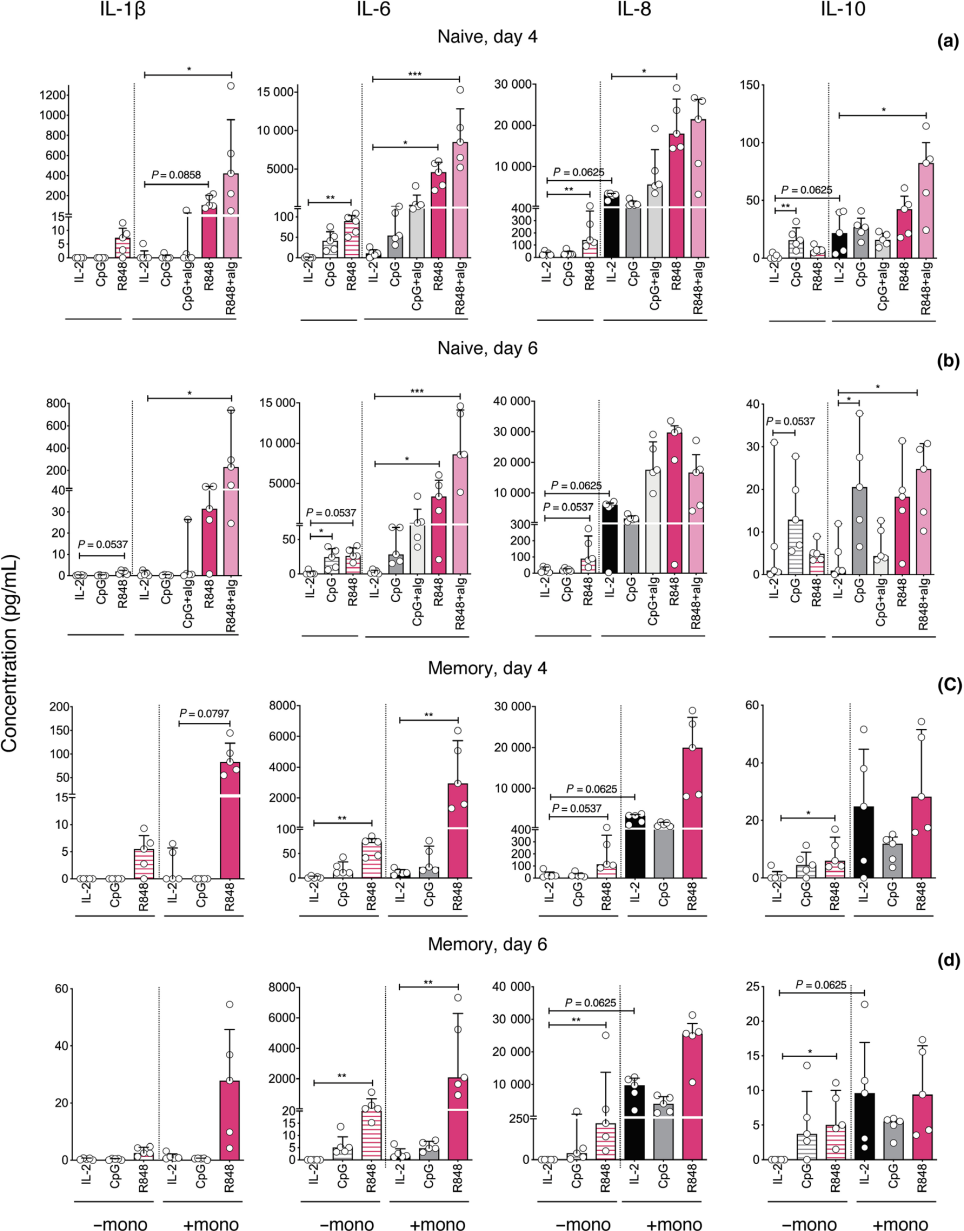
Increased detection of IL‐1β and IL‐6 when B cells are stimulated with R848 in the presence of monocytes for four and six days. Concentrations of IL‐1β, IL‐6, IL‐8 and IL‐10 in supernatants collected 4 and 6 days after culture of naive and memory B cells with stimuli as indicated, with and without monocytes. Results are shown for B cells from each donor (symbols, *n* = 5), and as medians and interquartile ranges for all donors. Asterisks indicate significance using Friedman and Wilcoxon tests, *P < 0.05, **P < 0.01, ***P < 0.001. Results of five different donors from three independent experiments.

Monocytes produce several cytokines in response to TLR7/8 or 9 signalling that support B‐cell differentiation, including IL‐1β, IL‐6, IL‐8, IL‐10 and TNF‐α.^11^, ^15^, ^16^, ^17^, ^18^ To examine whether these cytokines may mediate the effects of monocytes on B‐cell differentiation, we measured concentrations in the culture supernatants by cytometric bead array (CBA). Concentrations of all cytokines, except IL‐10 and TNF‐α, were higher in supernatants of R848‐simulated than in non‐stimulated or CpG‐stimulated naive B‐cells (Figure 7a and b, Supplementary figure 6d). Moreover, concentrations were substantially higher in R848‐stimulated cultures when monocytes were added, with consistent trends on days 4 and 6, and for both naive (Figure 7a and b) and memory B‐cells (Figure 7c and d). Concentrations of these cytokines were also higher in supernatants of R848‐stimulated naive and memory B cells cultured with monocytes without T cells, on both days 4 and 6 (Supplementary figure 6a–d). This indicates that monocytes produce these cytokines directly in response R848 and/or CD40L or induce their production by other cell types. IL‐10 and particularly IL‐8 and TNF‐α concentrations were higher in non‐stimulated cultures when monocytes were added, either with T cells or alone, indicating that these cytokines were constitutively produced by monocytes; nonetheless, IL‐8 secretion increased after R848 stimulation (Figure 7a–d, Supplementary figure 6a–d). Interestingly, TNF‐α concentrations were lower in cultures containing monocytes when T cells were also added, perhaps due to consumption by this cell type.^19^ In contrast, stimulation was required for robust detection of IL‐1β and IL‐6, as well for production to be enhanced by monocyte addition, consistent with studies elsewhere.^20^ Naive and memory B cells secreted IL‐6, IL‐8 and IL‐10, but not IL‐1β, when stimulated with R848 in the absence of other cell types (Supplementary figure 6a–c). Of note, monocyte addition had little effect on cytokine levels when cells were stimulated with CpG (Figure 7a–d): IL‐8 and IL‐10 levels were higher in CpG‐stimulated cultures containing monocytes, but not higher than in unstimulated cultures containing monocytes. Therefore, it is likely that upon stimulation via TLR8, monocytes produce IL‐1β and IL‐6, which promote B‐cell differentiation. IL‐1β and IL‐6 concentrations increased further when TLR stimulation via CpG or R848 was augmented with BCR stimulation, both on day 4 (Figure 7a) and on day 6 (Figure 7b). IL‐6 has established effects on B‐cell division^8^ and could explain why co‐stimulation via BCR promoted naive B‐cell division but not differentiation (Supplementary figure 5).

R848 stimulation induced CD14 and CD16 expression by monocytes recovered from our cultures (Supplementary figure 7). Similarly, others have found that monocytes that induce B‐cell stimulation after dengue infection are CD14^+^CD16^+^ 21 and that CD16^+^ monocytes are the main producers of IL‐1β and TNF‐α and also produce IL‐6 and IL‐8.^21^, ^22^ B cell‐activating factor (BAFF) and proliferation‐inducing ligand (APRIL) have been implicated in B‐cell stimulation by dengue‐infected CD14^+^CD16^+^ monocytes, and, like dengue infection, R848 stimulates BAFF and APRIL production by monocytes.^21^ BAFF and APRIL bind to BAFF family receptors that are expressed almost exclusively by B cells,^23^ including BAFF‐R and transmembrane activator and calcium‐modulator interactor TACI,^24^ as well as B‐cell maturation antigen (BCMA), which is mainly expressed by bone marrow plasma cells.^25^ Therefore, we examined whether BAFF‐R and TACI expression changes after B‐cell stimulation with R848, and whether these changes are altered by the presence of monocytes. Expression of BAFF‐R decreased and TACI increased as B cells transitioned from resting to activated and then plasmablast phenotypes (Supplementary figure 8a–c). This is consistent with reports that human naive B cells are TACI low and that CpG, but not anti‐Ig and CD40L, induces rapid TACI expression by activating the ERK1/2 pathway.^26^ The addition of monocytes had little or no effect on TACI and BAFF‐R expression by B cells during stimulation (Supplementary figure 8d). However, since R848 stimulation enhanced CD14 and CD16 expression by monocytes (Supplementary figure 7b and c), it is likely that the combination of R848 and monocytes provides optimal B‐cell differentiation because R848 induces TACI expression by B cells and, as shown by others, also induces BAFF and APRIL production by monocytes. Taken together, the results indicate that IL‐1β and IL‐6 are key mediators of B‐cell differentiation induced by engagement of TLR7/8 on both B cells and monocytes.

## Discussion

This study set out to identify markers that may be used to distinguish naive and memory B‐cells once activated. To this end, we sorted human naive and memory B cells and compared markers expressed following *in vitro* stimulation using a B‐cell activation protocol that had been optimised to generate ASCs. We found that naive and memory B cells remained phenotypically distinct for at least 6 days after activation, a time when we detected robust generation of ASCs from both subsets. It may therefore be possible to define whether B cells in acute *ex vivo* samples are naive‐ or memory‐derived, and whether memory B cells competitively inhibit naive B‐cell responses.^27^ Responding cells, identified using CD71, could be classified as naive‐ or memory‐derived using tSNE to combine multiple parameters including CD27, CD38, CD20, IgM and IgG expression and forward/side scatter. These findings expand upon previous studies showing that memory, but not naive, B‐cells upregulate CD38 in response to more minimal stimulation with CD40L and cytokines.^28^ This study also further refined the parameters required for *in vitro* differentiation of human B‐cells, including a key role for monocytes, discussed further below.

This study confirms that the TLR7/8 agonist, R848, is more potent than the TLR9 agonist, CpG, for inducing human B‐cell differentiation *in vitro*,^29^ and extends these findings to show that this is true for both naive and memory B‐cells. The greater potency of TLR7/8 compared to TLR9 agonists does not reflect TLR expression, since it is generally reported that B cells express higher levels of TLR9 than of TLR7 and that TLR9 is especially upregulated following BCR or CD40L.^6^, ^11^, ^30^, ^31^ Studies with mouse B cells similarly demonstrate that CD40L and TLR9 signalling stimulates B‐cell proliferation whereas CD40L and TLR7/8 signalling also stimulates differentiation.^32^ Moreover, co‐stimulation via the BCR prevents differentiation of mouse B‐cells, which are instead considered to enter the memory pathway.^32^ Here, when naive human B cells were co‐stimulated with anti‐Ig, they proliferated and upregulated CD27 but not CD38, reminiscent of a memory B‐cell phenotype. Similarly, Marasco *et al.* 9 showed that fewer ASCs were induced when anti‐Ig was added to a combination of CpG and CD40L, despite increased proliferation of both naive and memory B‐cells. 

Monocytes consistently enhanced B‐cell differentiation stimulated by R848 but not by CpG, and the effects of monocytes were more pronounced for naive than for memory B‐cells. Remarkably, we found that practically all memory B cells that underwent division when activated in the presence of monocytes alone became plasmablasts, but this was attenuated when T cells were also added. This indicates that the fate of memory B cells may depend upon the balance of innate versus adaptive signals present. Innate signals, which indicate the presence of foreign pathogens, may favor memory B‐cell differentiation into effector ASCs, whereas adaptive‐T cell responses, which tend to be more delayed, may favor a memory B‐cell fate. Naive B‐cells, on the other hand, appeared to require signals provided by both monocytes and T cells other than CD40L or IL‐21. The impact of monocytes on R848 but not CpG‐stimulated B‐cell differentiation concurs with the distribution of TLRs on human monocytes, which express TLR8 and, to a lesser extent, TLR7, but very little TLR9.^11^, ^14^ Accordingly, studies elsewhere demonstrate that monocytes are responsive to R848 but not CpG whereas B‐cells respond to both.^11^, ^33^ We therefore propose that monocytes required stimulation, in this case via R848 binding to TLR7/8, in order to enhance B‐cell differentiation *in vitro*. R848 induces monocytes to produce IL‐6 and IL‐1β, both of which have established roles in promoting B‐cell differentiation either by acting directly on B cells.^34^, ^35^ or through enhancing CD4^+^ T‐cell differentiation.^15^, ^34^, ^36^, ^37^ Indeed, co‐culture of B cells with monocytes alone indicated that R848‐stimulated monocytes were required for IL‐1β production using our activation protocol, and also substantially enhanced IL‐6 production. Others show that IL‐1β, produced by monocytes downstream of TLR signalling and inflammasome activation, induces differentiation of T follicular helper cells, which augment antibody production.^37^ Moreover, Ugolini *et al*.^38^ demonstrate that TLR8 engagement represents a mechanism via which CD14^+^CD16^+^ monocytes selectively detect viable as opposed to dead microorganisms, and signal this via producing IL‐1β, which activates T follicular helper cells. Similarly, Kwissa *et al.* 21 found that CD14^+^CD16^+^ monocytes, which are induced by dengue virus infection, and by R848 but not CpG, enhance B‐cell differentiation, providing another possible explanation for the differential role of monocytes in R848‐ versus CpG‐stimulated B‐cell differentiation. The capacity of TLR7/8 agonists to induce B‐cell differentiation is also apparent *in vivo* whereby adjuvanting inactivated influenza vaccine with a TLR7 agonist accelerates B‐cell differentiation and antibody secretion in mice^39^ and non‐human primate neonates.^40^ Analogously, inactivated whole‐virion influenza vaccine, as opposed to subunit or split virion vaccines, induces TLR7‐mediated B‐cell activation and increased antibody production in mice.^41^ IFN‐α is also secreted by monocytes and is essential for inducing TLR7 expression by B cells,^12^, ^42^, ^43^ but was not detected via CBA in any of our B‐cell culture supernatants (data not shown). The blockade of type I IFNs would help elucidate their impact in B‐cell differentiation. R848 also induced TACI expression by B cells, and it is known that TLR8 signalling induces BAFF and APRIL expression by monocytes.^21^ Further studies blocking the BAFF‐R and TACI receptors on the B‐cell surface in the presence and absence of monocytes are required to elucidate whether this pathway underlies monocyte help to naive and memory B‐cells. Taken together, these results indicate that the superior capacity of R848 over CpG to enhance B‐cell differentiation reflects both direct effects on B cells and indirect effects via CD14^+^CD16^+^ macrophage stimulation.

In conclusion, our findings demonstrate that monocytes are necessary for robust B‐cell differentiation and that R848, a TLR7/8 agonist, induces greater differentiation than CpG by stimulating not only B cells but monocytes, thereby potentiating IL‐1β and IL‐6 production. This work implicates monocytes as a potential mediator of the adjuvanting effects of TLR7/8 agonists on naive B‐cell responses and provides tools to better understand human B‐cell responses towards highly mutable RNA viruses.

## Methods

### Participants and ethics

Cells used in this study were from anonymous buffy coats [Australian Red Cross Blood Service (ARCBS), West Melbourne, VIC, Australia] or from heparinised venous blood of healthy volunteers who provided informed consent. The study was approved by the University of Melbourne Human Ethical Committee (ID 1443389.3 and 1443540) and the ARCBS Ethics Committee (ID 2015#8).

### Sample processing

PBMCs were isolated using Lymphoprep (STEMCELL Technologies, Vancouver, Canada) and Leucosep tubes (Greiner Bio‐One, Kremsmünster, Austria) according to the manufacturer’s instructions and either used immediately or cryopreserved. PBMCs were stained with CD19 BV421 (clone HIB19; Biolegend, San Diego, CA, USA), CD27 APC (clone O323; Biolegend), CD20 FITC (clone 2H7; BD, Franklin Lakes, NJ, USA) and a dump channel mix containing CD3 PE‐Cy7 (clone UCHT1; eBioscience, Waltham, MA, USA), CD10 PE‐Cy7 (clone HI10a; BD) and CD14 PE‐Cy7 (clone M5E2; BD). Naive B‐cells, memory B‐cells, non‐B lymphocytes and monocytes were sorted with a FACSAria III sorter (BD Biosciences) with gating as shown in Figure 2a.

### B‐cell culture and stimulation

PBMCs and sorted B‐cell subsets were stained with Invitrogen™ CellTrace™ CFSE (Thermo Fisher, Waltham, MA, USA) to track B‐cell division. Briefly, cells were resuspended at 1 × 10^7^ mL^−1^ in PBS containing 0.1% BSA and 1 µL mL^−1^ CFSE and incubated for 10 min at 37°C before washing twice with RPMI containing 10% FCS. Sorted B‐cell subsets were mixed with unlabelled non‐B lymphocytes, and also with unlabelled monocytes where indicated. The number of each cell type was calculated to yield mixtures containing 10% B cells and, when present, 5% monocytes. Four different cell mixtures were made for each donor, that is naive B‐cells + T‐cells; naive B‐cells + T‐cells + monocytes; memory B‐cells + T‐cells; and memory B‐cells + T‐cells + monocytes. PBMCs and sorted cell mixtures from each donor were used at a concentration of 2 × 10^6^ cells mL^−1^ in complete media (RPMI containing 9% FBS, 2 mm l‐glutamine, 1 mm MEM sodium pyruvate, 100 μm MEM non‐essential amino acids, 5 mm HEPES buffer, 55 μm 2‐mercaptoethanol, 100 u mL^−1^ penicillin, 100 μg mL^−1^ streptomycin). A total of 1 × 10^6^ PBMCs or 0.5 × 10^6^ mixed sorted cells were distributed into each of up to five wells of a 48‐well plate. Unstimulated control wells also contained recombinant human IL‐2 at 10 U mL^−1^ (Roche Diagnostics GmbH, Mannheim, Germany). Cells in all other wells were stimulated with recombinant human IL‐21 (eBioscience) at 50 ng mL^−1^, and recombinant human soluble CD40 ligand/TRAP (sCD40L; Peprotech, Rocky Hill, CT, USA) at 1 µg mL^−1^, and either CpG oligonucleotide type B at 5 µg mL^−1^ (InvivoGen, San Diego, CA, USA) or Resiquimod ≥ 98% HPLC (R848; Sigma Aldrich, Saint Louis, MO, USA) at 1 µg mL^−1^, both with and without the F(ab’)2 component of goat anti‐human IgG/A/M at 10 µg mL^−1^ (Cat# 109‐006‐064; Jackson ImmunoResearch, West Grove, PA, USA).

### Post‐activation analysis

Cells were harvested after 4 and 6 days of incubation at 37°C in 5% CO_2_. At each time point, 120 000 cells from the wells containing PBMCs and 100 000 cells from the wells containing sorted B‐cell subsets were collected to perform a dual‐colour ELISPOT assay, and the remaining cells were stained for flow cytometry analysis. Supernatants from each well were also collected to perform CBA analysis.

### Flow cytometry

To determine the phenotype of B‐cells post‐stimulation, cells were stained with LIVE/DEAD™ Fixable Aqua Dead Cell Stain Kit (Thermo Fisher, Waltham, MA, USA) and then with anti‐human IgM BV605 (clone MHM88; Biolegend), IgG BV786 (clone G18‐145; BD), CD27 APC (clone 323; Biolegend), CD20 APC‐H7 (clone 2H7; BD), CD38 PE (clone HIT2; BD), CD19 PE‐CF594 (clone HIB19; BD), CD71 PE‐Cy7 (clone CY1G4; Biolegend), IgD BUV395 (clone IA6‐2; BD) and CD21 BUV737 (clone B‐Iy4; BD). The dump channel mix consisted of BV510‐labelled anti‐human CD3 (clone OKT3), CD10 (clone HI10a), CD14 (clone M5E2) and CD16 (clone 3G8), all from Biolegend. Samples were stained according to standard techniques and fixed briefly with 1% formaldehyde before acquiring data on an LSRFortessa flow cytometer (BD Biosciences). Data were analysed using FlowJo v10.5.3 (Tree Star, Inc., Ashland, OR, USA).

### Dual‐colour ELISPOT analysis

Harvested cells were counted and transferred to 96‐well ELISPOT plates (Merck Millipore, Darmstadt, Germany) that had been pre‐coated with 10 µg mL^−1^ of unconjugated goat anti‐human IgA, IgG and IgM polyclonal antibodies (Jackson ImmunoResearch). Cells were added at a range of concentrations, that is 4 × 10^4^, 2 × 10^4^ and 1 × 10^4^ cells 100 µL^−1^, in complete media and incubated for 5 h at 37°C in 5% CO_2_. Cells were washed off before incubating with a mix of alkaline phosphatase‐conjugated AffiniPure Anti‐human IgG Fcγ and horseradish peroxidase‐conjugated AffiniPure Anti‐human IgM Fc5μ, both at 1 µg mL^−1^, for 2 h at room temperature. AP (AP Conjugate Substrate Kit; Bio‐Rad, Hercules, CA, USA) and then peroxidase substrate (BD ELISPOT AEC Substrate Set; BD) were successively added and washed with water to reveal red and then blue spots, respectively. Spots were counted with an AID EliSpot/FluoroSpot Reader (Autoimmun Diagnostika GmbH, Straßberg, Germany) using two colour settings.

### Cytometric bead array

Culture supernatants were assessed using a human CBA kit (BD) to detect IL‐1β, IL‐6, IL‐8, IL‐10, IFN‐α and TNF‐α.

### Statistical analysis

Significance was assessed using Wilcoxon signed‐rank test for non‐parametric distributions, or paired *t*‐test when values were normally distributed. For multiple comparisons, a Friedman test was used. Analysis was performed using SPSS (IBM, Armonk, NY, USA) and Prism (GraphPad, San Diego, CA, USA) software.

## Conflict of interest

The authors declare no conflict of interest.
