# Supplementary material for: Distinguishing naive‐ from memory‐derived human B cells during acute responses
Source: Clin Transl Immunology. 2019 Nov 13;8(11):e01090. doi: 10.1002/cti2.1090 (PMC6851823; doi:10.1002/cti2.1090)
Supplement: Supplementary file 1 [file CTI2-8-e01090-s001.pdf]

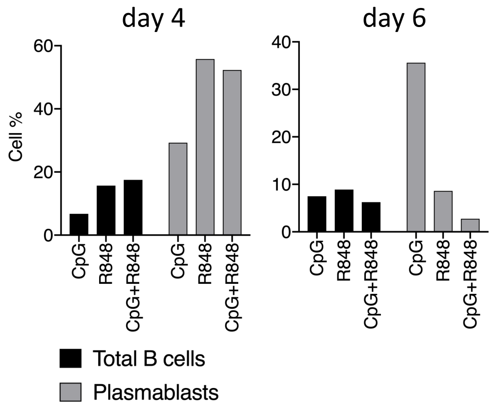

Supplementary figure 1. Preliminary experiment comparing stimuli for human B cell activation. PBMCs from a single donor were cultured with IL-21 and sCD40L and either CpG or R848 alone, or both combined for 4 and 6 days before determining percentages of total cells that are B cells and percentages of B cells that are plasmablasts, as described in Figure 1.

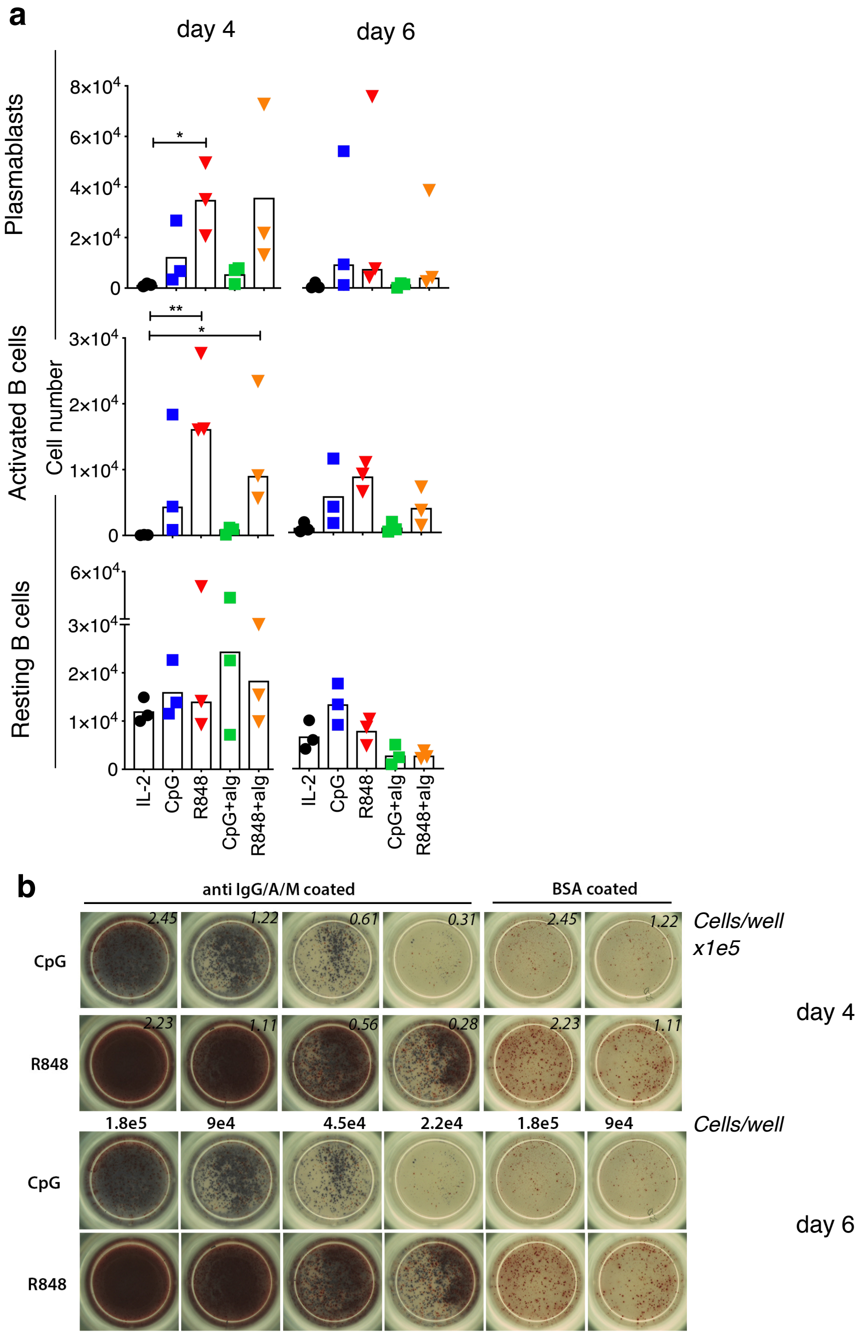

Supplementary figure 2. Comparison of stimuli for human B cell activation. (**a**) PBMCs were cultured with IL-21 and sCD40L combined with either CpG or R848 alone, or also with anti-human Ig for 4 and 6 days before quantifying the number of B cells with plasmablast or resting or activated B cell phenotypes, as described in Figure 1. Results are shown for individual donors (symbols, n=5), and as medians (bars). Asterisks indicate stimuli that had a significant effect on numbers compared to the IL-2 control using Friedman test, * $p<0.05$ ** $p<0.01$. (**b**) ASC ELISPOT images of B cells from total PBMC that have been stimulated with sCD40L, IL-21 and either CpG or R848 for 4 and 6 days then incubated on ELISPOT plates for 5h to detect cells secreting IgM (red spots) and IgG (blue spots).

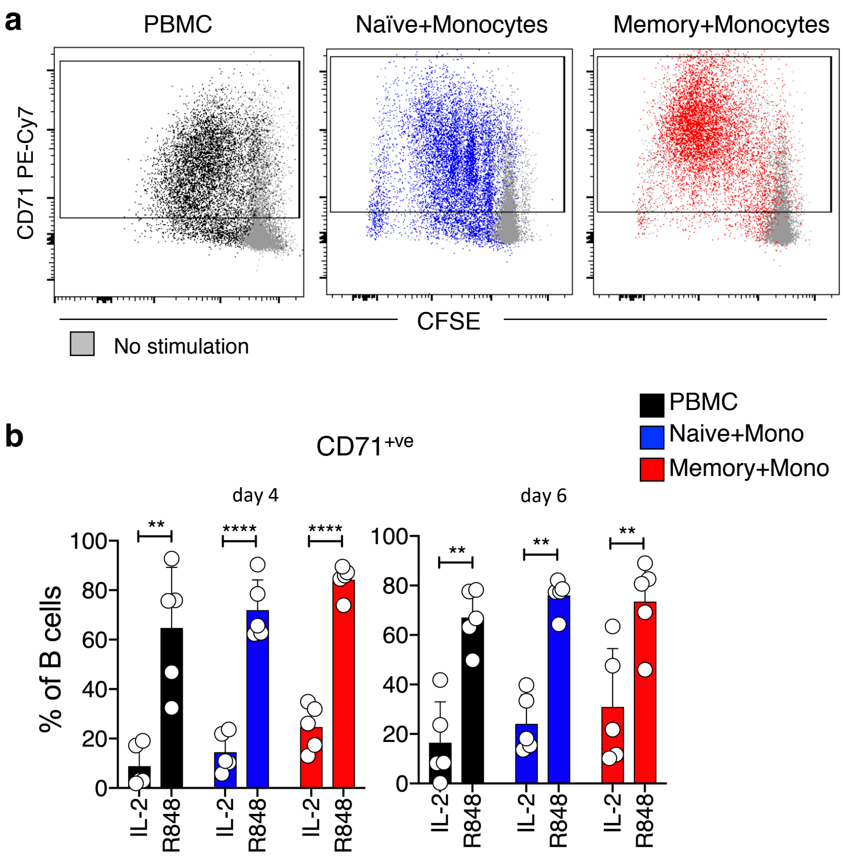

Supplementary figure 3. CD71 expression increases after B cell stimulation. (a) PBMCs and sorted naive and memory B cells from a representative donor were cultured for 4 days with and without R848 before assessing B cell division versus CD71 expression. (**b**) Percentages of B cells that are CD71^{+} are shown for individual donors (symbols, n=5), and as means with standard deviations for all donors (bars) with asterisks indicating significance using paired t-Test , ** p<0.01, ****p<0.0001.

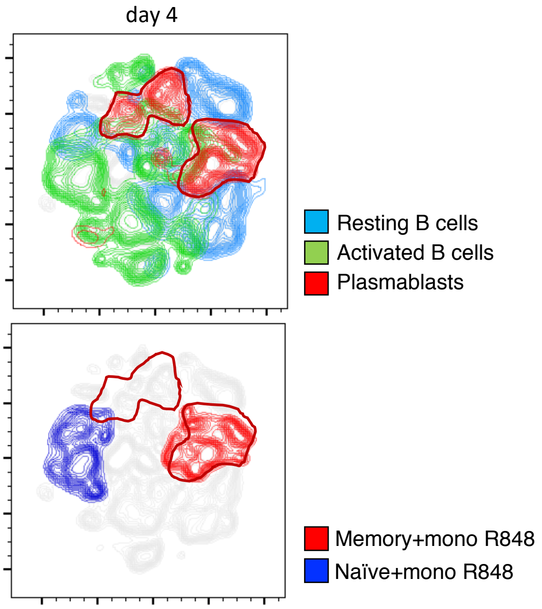

Supplementary figure 4. Naive and memory B cells are discernible at early time points after activation. tSNE plots show clustering of cells within the B cell gate based on all markers assessed and light scatter. Results represent concatenated data for naive and memory B cells from all donors assessed 4 days after stimulation with R848 with monocytes. Plots are overlaid with either the pre-defined B cell analysis gates based on CD27 and CD38 expression (top panels) or with B cell subset (bottom panels). Note that gaps in the plasmablast region reflect the location of memory B cells stimulated with R848 in the absence of monocytes.

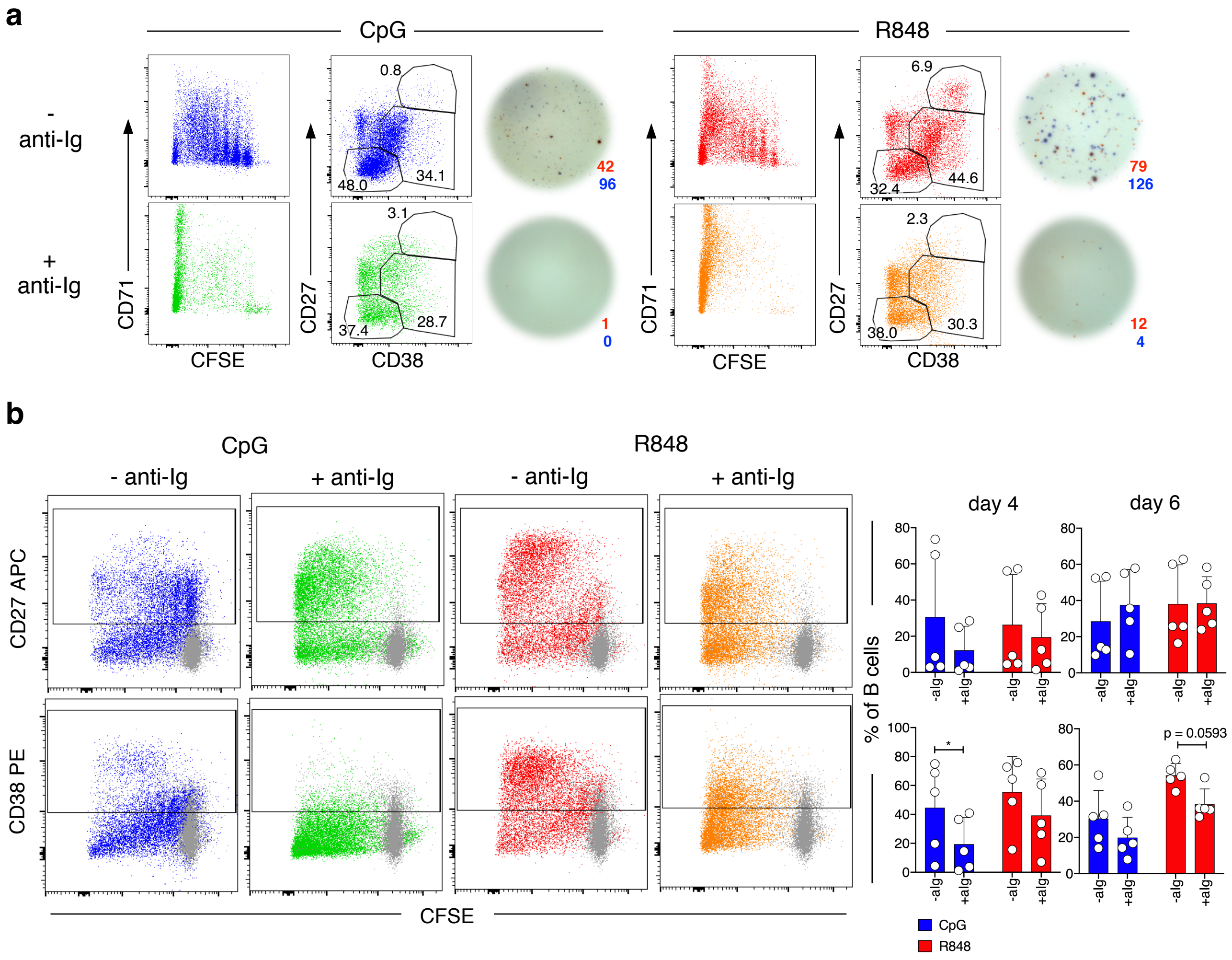

Supplementary figure 5. BCR stimulation via anti-Ig increases division but reduces differentiation into plasmablasts when naive B cells are stimulated via TLRs in the presence of monocytes. (**a**) Results are shown for naive B cells from a representative donor, assessed 6 days after stimulation with R848 with and without anti-Ig. Division is indicated by decreasing CFSE and increasing CD71 expression whereas differentiation is indicated by increasing CD27 and CD38 expression, and by detection of ASC secreting IgM (red spots) or IgG (blue spots) via ELISPOT. (**b**) CD27 and CD38 expression change with each division after 6 days of CpG and R848 stimulation without and with anti-Ig for a representative donor. Proportions of CD27^{+} and CD38^{+} B cells for each donor (n = 5) and condition are summarized as means and standard deviations. Asterisks indicate significance using paired t-Test, * $p<0.05$

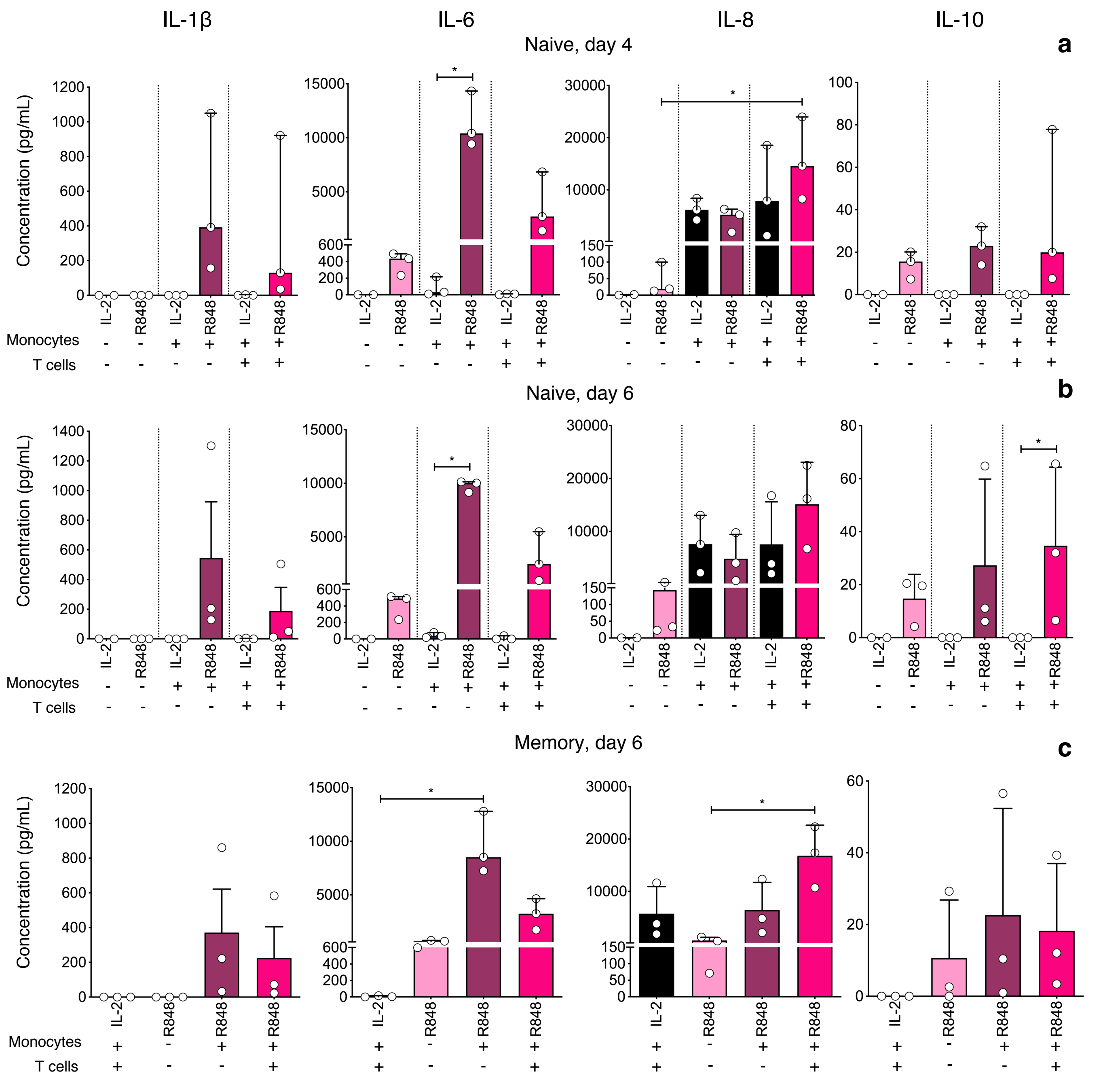

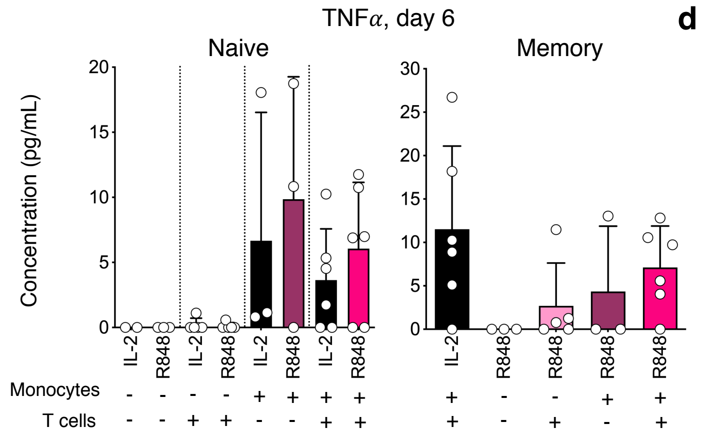

Supplementary figure 6. Increased detection of IL-1β, IL-6 and IL-10 when B cells are stimulated with R848 in the presence of monocytes and absence of T cells. (**a-c**) Concentrations of IL-1β, IL-6, IL-8 and IL-10 in supernatants collected 4 days after culture of naive B cells and on day 6 of naive and memory B cells with stimuli as indicated, with and without monocytes and T cells. (**d**) Concentrations of TNFα in supernatants collected 6 days after culture of naive and memory B cells with stimuli as indicated, with and without monocytes and/or T cells. Results are shown for B cells from each donor (symbols, n = 3 (a-c); n = 3 for cultures with B cells alone or B cells and monocytes, n = 5 for cultures with B cells and T cells, and n = 6 for cultures with B cells, monocytes and T cells (d)) and as medians and interquartile ranges for all donors. Asterisks indicate significance using Friedman test, $_* P < 0.05$.

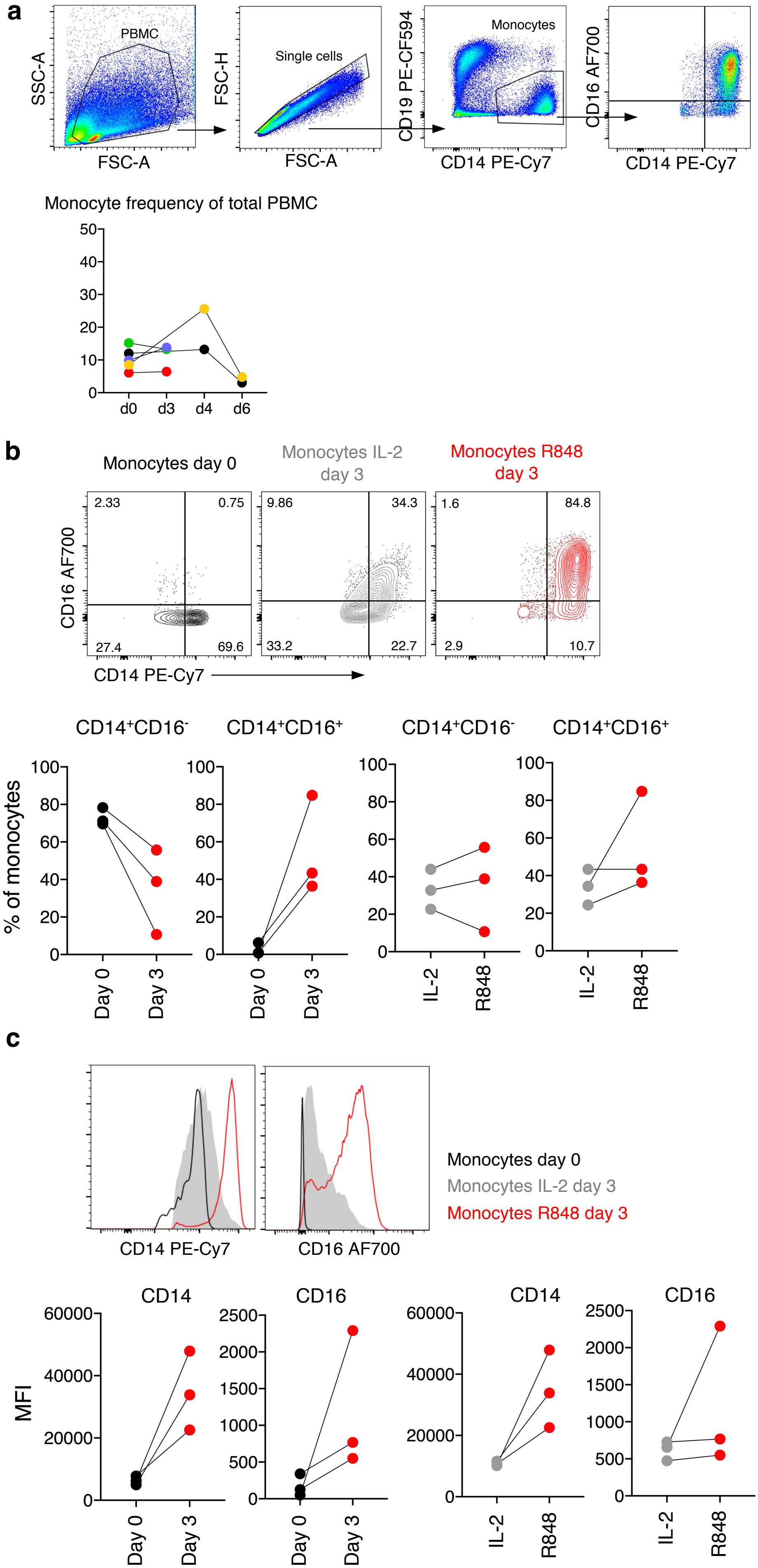

Supplementary figure 7. Monocytes differentiate to CD14⁺CD16⁺ cells after stimulation with R848. (**a**) Monocyte analysis gates and frequency of monocytes before and 3, 4 and 6 days after culture. Each donor is represented in a different colour. (**b**) FACS profiles of monocytes from a representative donor at baseline and at day 3 of stimulation with R848 (top panel). Percentages of monocytes in the analysis gates on days 0 and 3 post-stimulation with R848 (bottom left panel) and on day 3 post-culture with IL-2 or R848 (bottom right panel) are shown for individual donors (symbols, n = 3). (**c**) Monocyte expression of CD14 and CD16 on day 0 and on day 3 post-culture with IL-2 or R848 (top panel). Mean fluorescence intensity (MFI) of CD14 and CD16 on monocytes on days 0 and 3 post-stimulation with R848 (bottom left panel) and on day 3 post-culture with IL-2 or R848 (bottom right panel) are shown for individual donors (symbols, n = 3).

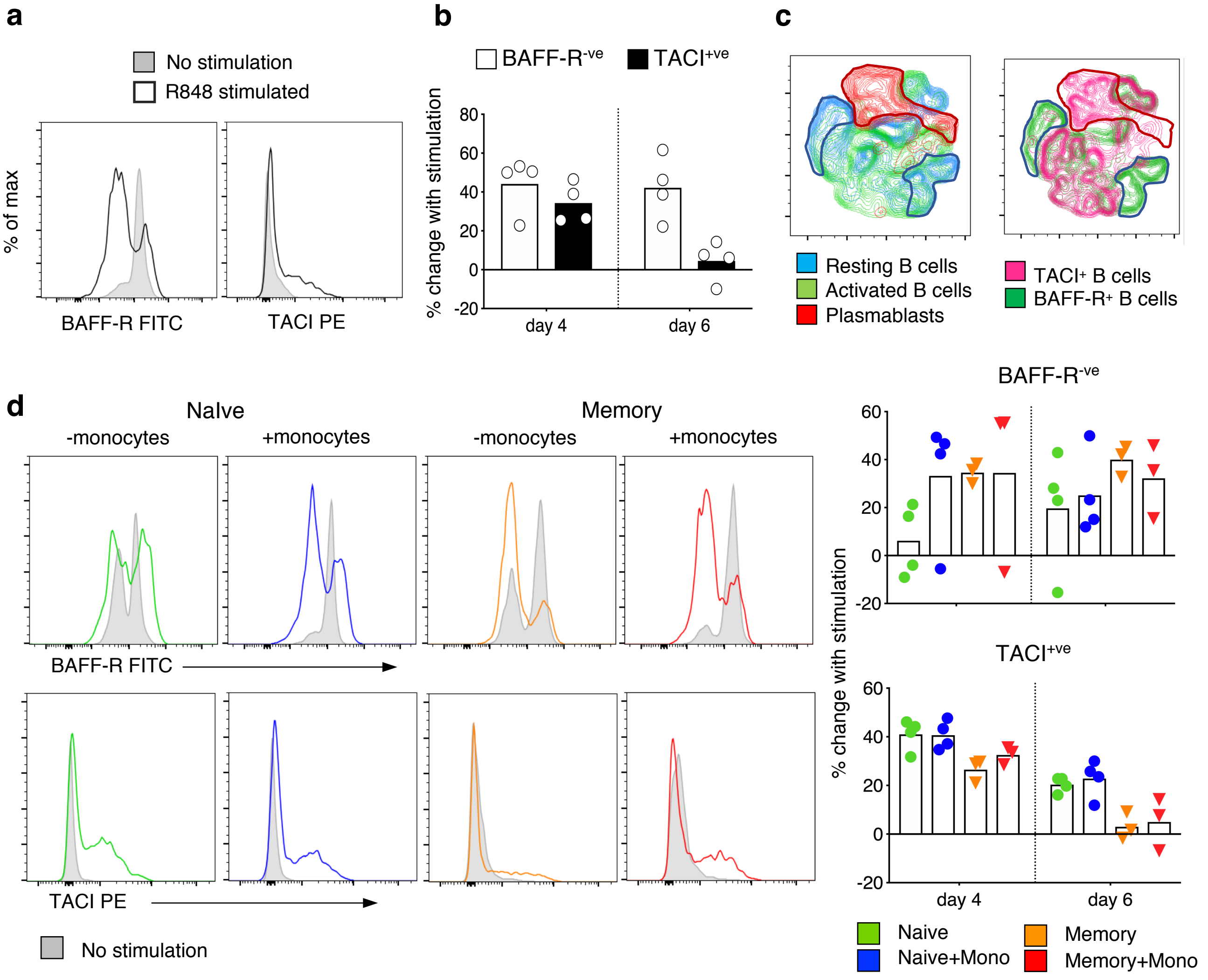

Supplementary figure 8. BAFF-R and TACI expression are modulated when B cells are stimulated via TLRs leading to plasmablast formation with minimal effects of adding monocytes. (a) BAFF-R and TACI expression by B cells within PBMCs from a representative donor that have been cultured for 6 days with IL-2 (no stimulation) or with R848, sCD40L, and IL-21. (**b**) Results for four individuals are summarized as median percentage change in expression of BAFF-R and TACI, calculated as percentage in R848 stimulated minus percentage without stimulation. (**c**) tSNE plots, presented as per Figure 1, have been overlaid with the pre-defined B cell analysis gates (left panel) or with the or TACI^{+} and/or BAFF-R^{+} populations. (**d**) BAFF-R and TACI expression by B cell subsets that have been cultured for 6 days with and without R848 stimulation and with and without monocytes. Data for four donors is represented as concatenated FACS profiles and as percentage change in expression, as in B.
